# Research on dust control technology and numerical simulation of conical guiding air curtain in fully mechanized excavation face

**DOI:** 10.1038/s41598-024-63881-4

**Published:** 2024-06-06

**Authors:** Xin Meng, Qiqiang Gao, Jie Li, Guoan Zhao

**Affiliations:** Shaanxi Yongming Coal Mine Co., Ltd., Yan’an, 717300 China

**Keywords:** Excavation working face, Dust, CDAC device, Guiding wind curtain, Dust control effect, Engineering, Energy infrastructure

## Abstract

The dust pollution caused by the operation of fully mechanized heading face poses a serious threat to the safety production of operators and working face. To reduce dust concentration at the fully mechanized heading face, this study analyzed dust samples collected from various positions to understand the particle size distribution characteristics. Based on these findings, a conical diversion air conditioning (CDAC) device was designed to create a radial air curtain for dust control in the roadway cross-section. Computational Fluid Dynamics (CFD) was then employed to investigate the airflow and particle dynamics when the cone-shaped deflector was in closed and open states. The results show that in the fully mechanized heading face, the dust distribution in the working area of the roadheader driver is relatively dense, and the dust particles with particle size ≤ 8 μm account for a large proportion. When the CDAC device is deployed, the axial airflow in the roadway is changed into a rotating airflow along the roadway wall, and an air screen is established in the working area of the roadheader driver to block the outward diffusion of dust. When the pressure air outlet is arranged 30 m away from the tunneling head, the pressure air volume is set to 400 m^3^/min, and the CDAC device can better form the air curtain barrier to block the dust particles. It provides a new method for effectively controlling the dust concentration of the fully mechanized heading face and directly ensuring the health of the roadheader driver.

## Introduction

At present, coal is one of the important energy sources for social development and industrial progress. With the continuous increase of coal seam mining depth, the degree of mechanization and automation of mines has gradually increased, and the dust pollution problem of fully mechanized working face has become increasingly prominent^1–3^. When the fully mechanized working face does not take any preventive measures, the dust concentration can reach 60,000 mg/m^3^. Even if certain measures are taken, the dust concentration can still be as high as 1200–1300 mg/m^3^, far exceeding the safety range required for underground mine mining^4^. When high-concentration dust particles diffuse in mines or roadways, they not only have serious explosion hazards, but also greatly increase the risk of pneumoconiosis among mine workers^5^. Long-term exposure of mine workers to high-concentration dust environment can cause incurable fibrous lesions in lung tissue, namely pneumoconiosis, which makes infected workers need lifelong medical care^6^.

**Figure 1:**
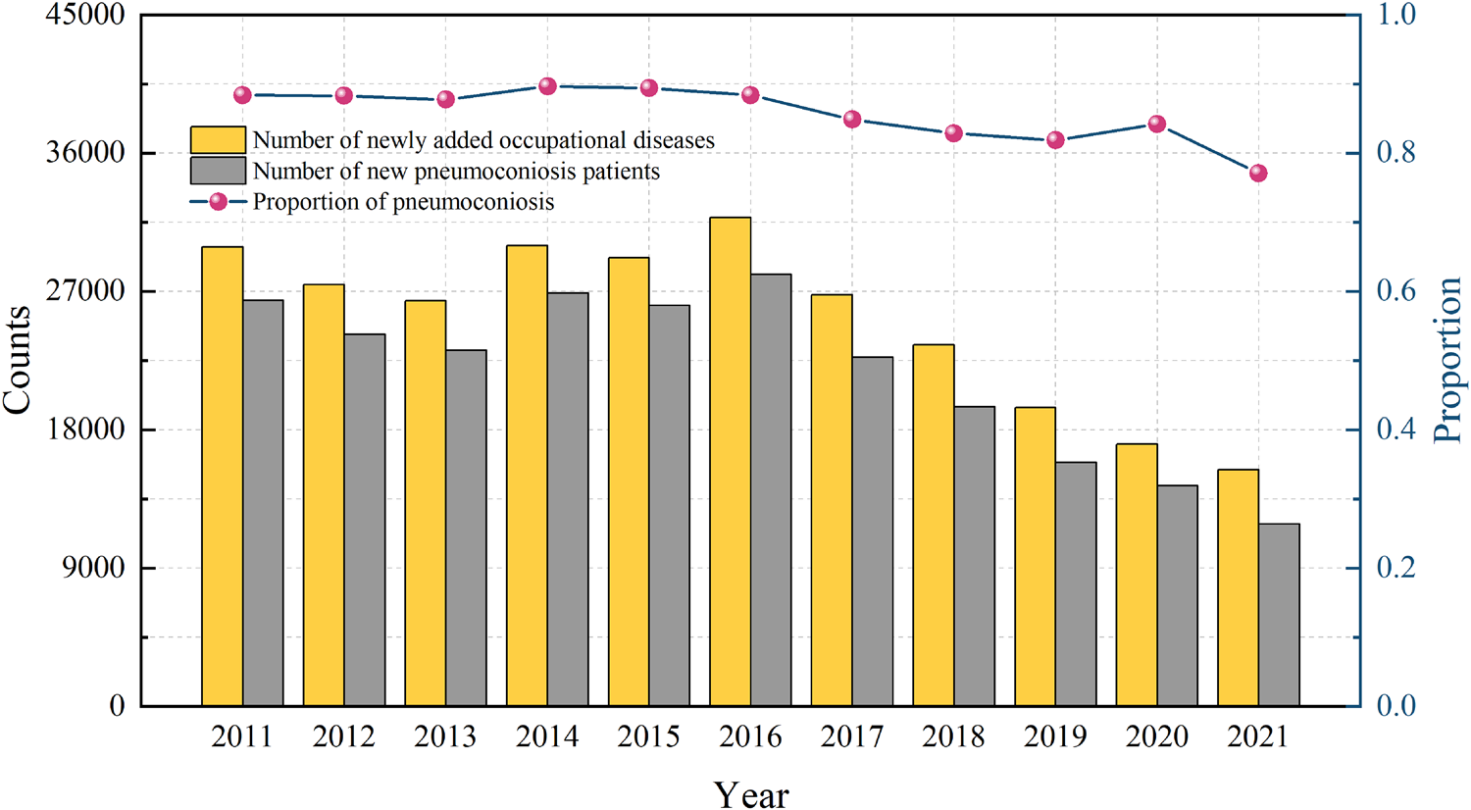
The number and proportion of occupational diseases in China from 2011 to 2021.

China is rich in coal resources, and coal energy still occupies the main position of energy consumption structure^7^. In the process of coal resource exploitation, a large amount of dust will inevitably be produced, and the incidence of pneumoconiosis will be greatly increased. Pneumoconiosis accounts for more than 80% of new occupational cases in China, far exceeding other occupational diseases^8,9^. The National Health Commission of China makes statistics on the number of occupational diseases every year^10^, as shown in Fig. 1 in the past 10 years. According to Fig. 1, although the number of new cases of pneumoconiosis in China has gradually decreased, pneumoconiosis is still one of the most harmful occupational diseases in the world. Therefore, dust pollution control during mining is still a problem that needs to be solved at this stage.

As an important part of coal mining, the dust control of fully mechanized heading face has also attracted much attention. Most researchers have optimized the dust suppression performance of the roadway auxiliary ventilation system by improving the air supply volume, air duct length and other parameters of the roadway auxiliary ventilation system^11–13^. Geng et al.^14^ used Euler–Euler and Euler–Lagrange methods to investigate the diffusion characteristics of dust particles under the conditions of particle size and initial wind speed in typical coal roadways in the auxiliary ventilation system. It shows that when the initial wind speed is high, the stratification of particles with different particle sizes in the dust is more obvious. Yu et al.^15^ analyzed the mechanism of dust diffusion pollution under forced exhaust ventilation in fully mechanized heading face by CFD-DEM coupling. Combined with field measurement, numerical simulation and physical experiment, Hua et al.^6^ analyzed the temporal and spatial evolution law of dust pollution under the condition of blower ventilation and long pressure short pumping ventilation, and obtained the optimal distance to effectively control dust during the excavation of fully mechanized excavation face. In order to effectively suppress and prevent dust pollution, some researchers have studied the methods of dust prevention and control in coal mines. Wang et al.^16^ studied the flow field characteristics inside the nozzle and near the nozzle outlet under different air supply pressures in the roadway model through numerical simulation and physical experiments, and obtained the change trend of dust suppression performance parameters with the change of air supply pressure. Wang et al.^17^ put forward the integrated dust removal technology of foam and water mist. According to the characteristics of dust production in tunneling operation, a new type of flat fan foam nozzle was designed to realize the multi-stage atomization effect of air and water, effectively improve the dust removal effect and greatly reduce the dust removal cost. Liu et al.^18^ designed an integrated vortex dust removal system based on the swirling jet theory. The dust removal effect of the system was analyzed and tested by numerical simulation and field experiments. The results show that the designed system has better control and removal effect for smaller dust particles. The concentration of total dust and inhaled dust in the air is reduced to 10 mg/m^3^.

In summary, while dust control technologies for fully mechanized heading faces have made significant progress, current research primarily focuses on the cutting position of the road header rather than directly ensuring the health of the road header operators. To effectively reduce dust concentration in the heading face, it is essential to understand the dust particle distribution characteristics at different positions within the heading face. This study collected dust samples from various locations within the fully mechanized heading face and used image analysis techniques to determine the particle size distribution characteristics at these positions. Based on these characteristics, a CDAC device was designed to create a radial air curtain for effective dust control across the roadway cross-section. Numerical simulations were then conducted to explore the optimal minimum distance of the air outlet when the deflector device is closed and the optimal airflow rate when the deflector device is open. The simulation results were validated against field data. This study provides a new method for effectively controlling dust concentration in fully mechanized heading faces and directly safeguarding the health of road header operators.

## Distribution characteristics of dust particles in fully mechanized heading face

Dust is not only a kind of solid microparticles that can float in the air for a long time, but also a kind of dispersion system called aerosol. The dispersed phase is solid microparticles, and the dispersion medium is air^19–21^. Dust is defined from the perspective of explosion, and generally refers to rock particles with a particle size (the average cross-sectional diameter of the dust particles) of 0.75–1 mm or less^22,23^; rock powder is defined from the perspective of industrial hygiene, which generally refers to rock dust particles with particle size below 10–45 μm^24,25^.

A large amount of dust emitted during the production process is called productive dust^26,27^. Mine dust belongs to a type of productive dust, which is the general term of various rock and mineral particles produced in the process of mine construction and production. Coal mine dust is the general term of coal dust, dust and other toxic and harmful dust. In addition, there are a small amount of metal particles, artificial organic dust (such as soot) generated during blasting and artificial inorganic dust (such as cement dust) generated during arching and shotcrete construction in coal mines^28^.

### Dust sampling in production process

**Figure 2:**
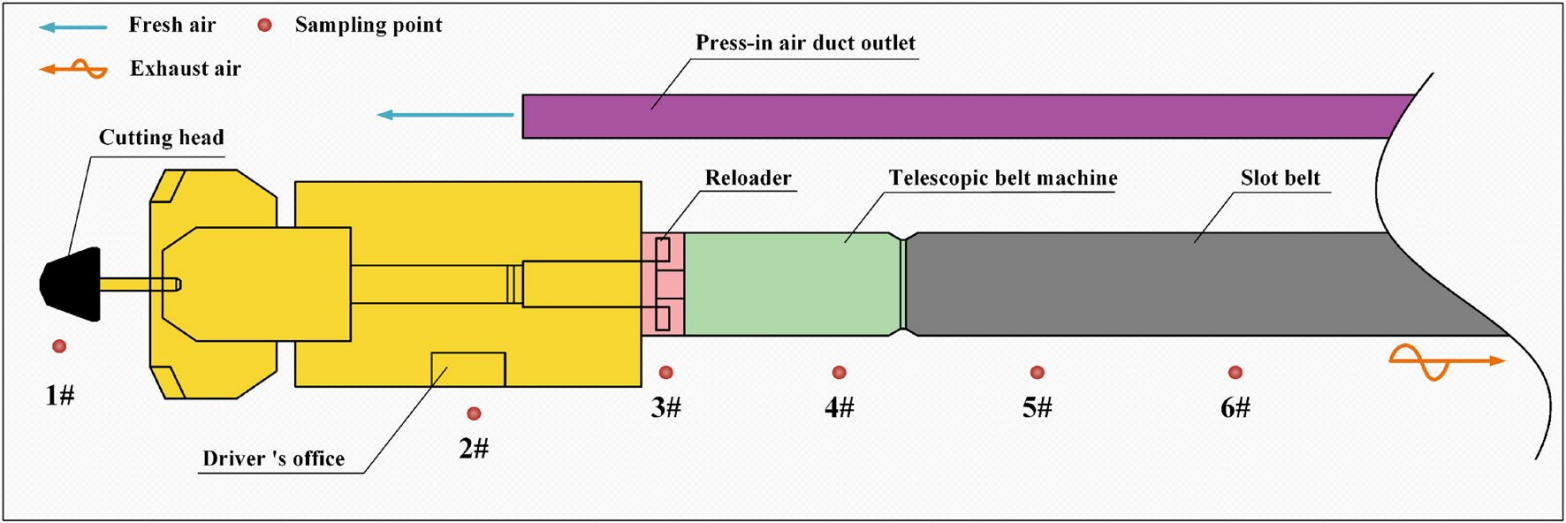
Dust sampling point layout diagram of fully mechanized excavation face. 1# sampling point is at the mining position of the heading face; 2# sampling point is at the location where the operator controls the heading machine; 3# sampling point is on the leeward side of the transfer machine, which is responsible for transferring the rock fragments generated at the heading face; 4# sampling point is on the leeward side of the telescopic belt conveyor, which is responsible for transporting the rock fragments; 5# sampling point is 100 m from the heading face; and 6# sampling point is 200 m from the heading face.

During actual production at the Yongming Coal Mine heading face, fresh air is supplied to the heading face through a forced ventilation system. This ensures that dust particles generated at the heading face are expelled from contaminated air, thereby achieving dust suppression. Consequently, dust samples are collected from various production processes at the fully mechanized heading face. According to the dust measurement points specified by the "Coal Mine Safety Regulations," critical positions at the heading face are sampled. Six sampling points are set up at the fully mechanized heading face: the heading face (1#), the operator's position (2#), the leeward side of the transfer machine (3#), the leeward side of the telescopic belt conveyor (4#), 100 m from the heading face (5#), and 200 m from the heading face (6#). The specific sampling locations are shown in Fig. 2. Each sampling point is measured three times per sampling operation.

### Determination method of dust particle size distribution

**Figure 3:**
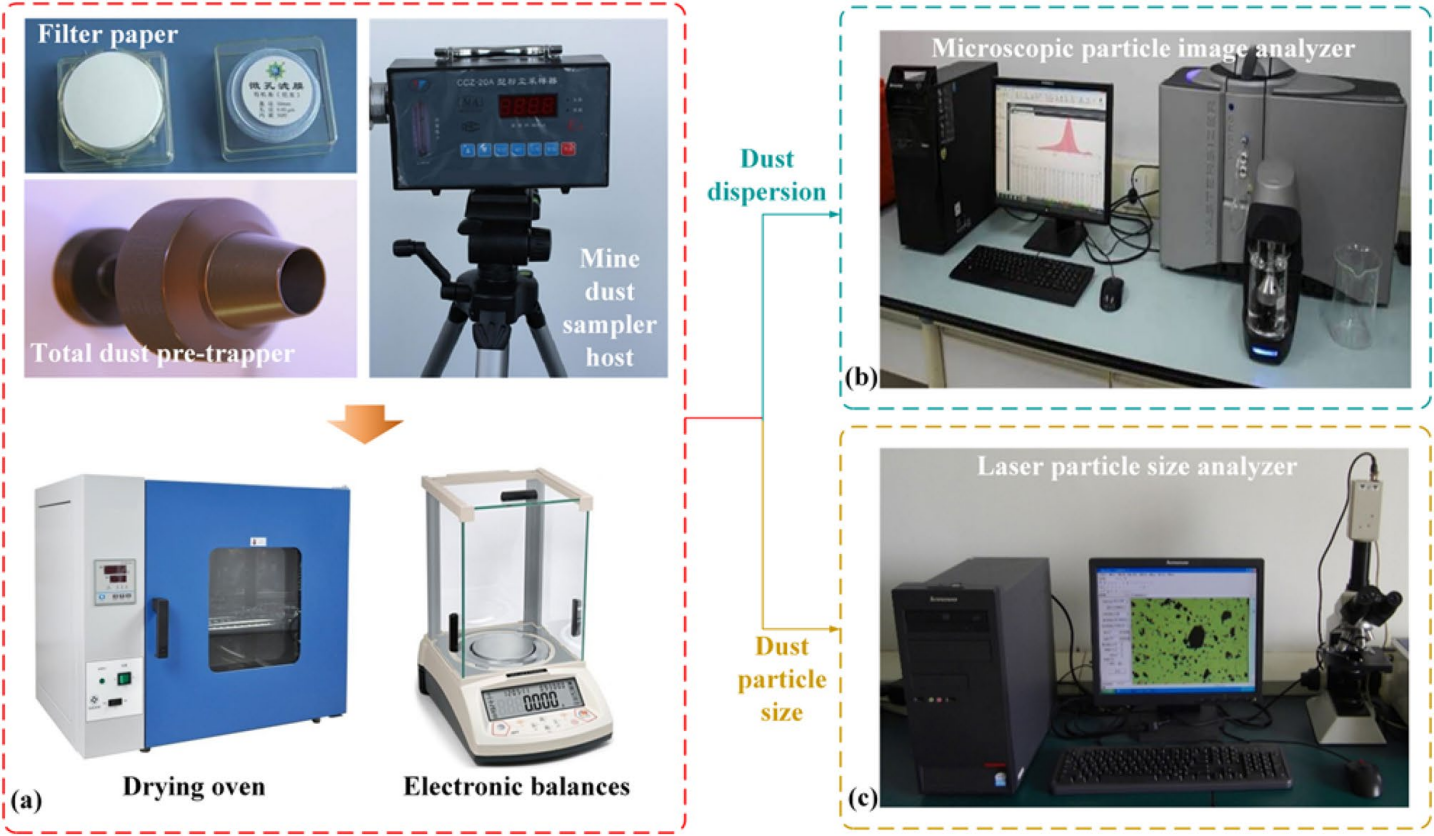
Schematic diagram of the method for measuring the physical and chemical properties of dust in fully mechanized excavation face. (**a**) Experimental pre-processing; (**b**) microscopic particle image analyzer; (**c**) laser particle size analyzer.

The physical and chemical characteristics of dust in fully mechanized heading face mainly include dust concentration, particle size, dispersion and other parameters. The specific measurement method is shown in Fig. 3. Dust concentration was measured using a full-dust pre-trap in the mine dust sampler (CCZ-20A) to form dust samples. Before collecting samples, select the sampling location and securely fix the dust sampler horizontally on the tripod platform. Install the pre-collector with the filter (Φ 75 mm or Φ 40 mm perchloroethylene fiber filter) firmly on the sampler head connector, ensuring the pre-collector's inlet is positioned within the dust-laden airflow. When collecting samples, the sampling time is preset according to the type, concentration and operation of dust on site. Generally, the sampling time is 20–25 min, and the place with higher dust concentration is generally preset for 2–5 min. After the sample collection, remove the filter membrane and gently place it in the corresponding sample box. Dry the filter membrane using a drying oven, and after the process is completed, weigh it using an electronic balance and record the weight.

In order to further understand the actual situation of dust particle size distribution in each process of fully mechanized heading face, the proportion of respirable dust in the dust generated on site was investigated. Dust was obtained on the site of fully mechanized heading face in Yongming Coal Mine, and the frequency distribution data of particles (the percentage of each particle size interval) were obtained by laser particle size analyzer (Mastersizer 3000). The role of the microscopic particle image analyzer (Winner 99) is to use the image processing software to perform a series of processing on the collected original image, and then the particle size analysis data can be obtained.

### Analysis of dust particle size distribution characteristics of each process

**Figure 4:**
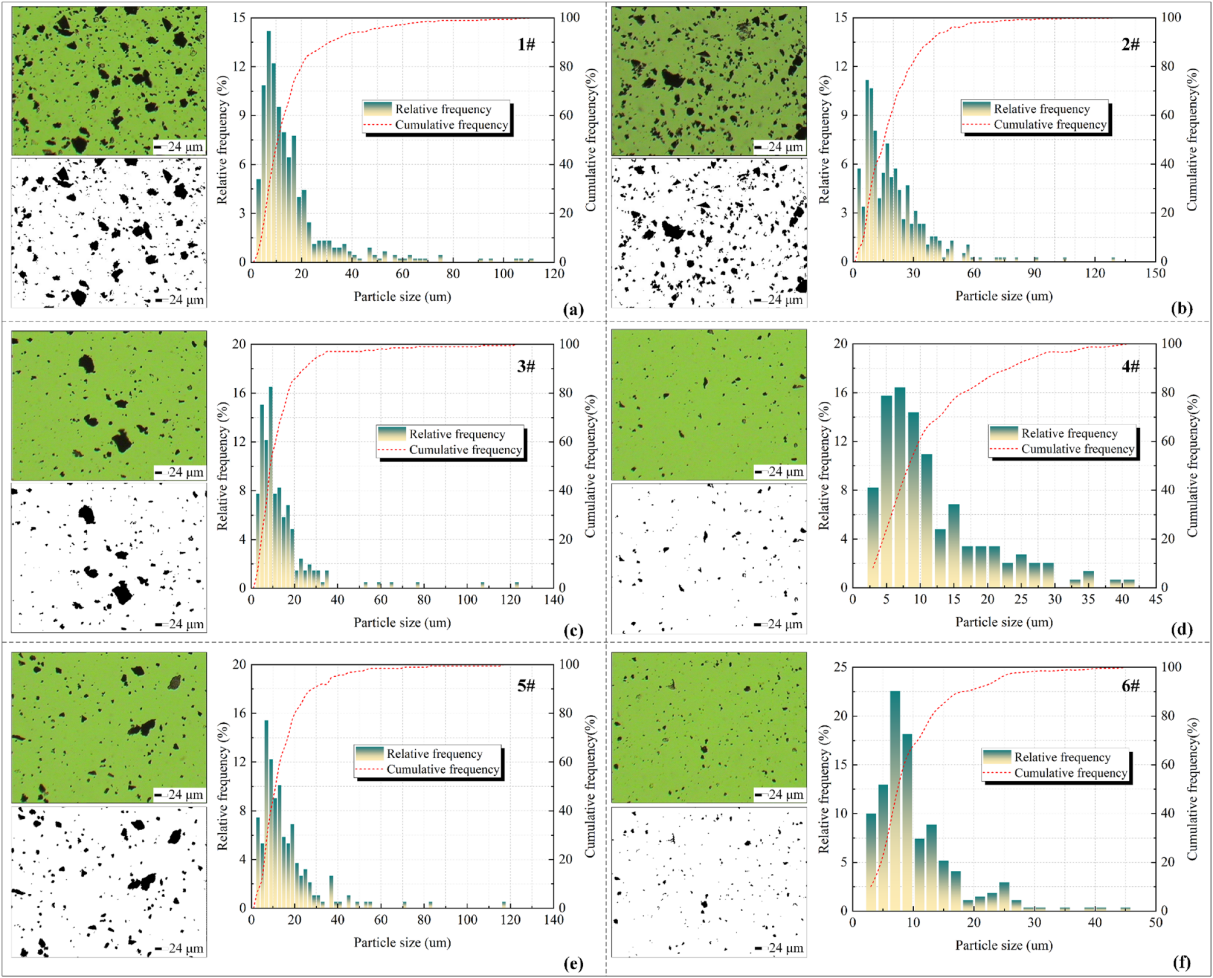
Grain size analysis diagram of each process in fully mechanized excavation face. (**a**) Heading face; (**b**) Driver 's office; (**c**) The downwind side of the loader; (**d**) The downwind side of the telescopic belt conveyor; (**e**) 100 m from the heading face; (**f**) 200 m away from the heading face. The green image represents the microscopic particle image, the black and white image depicts the binarized particle image, and the histogram illustrates the frequency distribution of particle diameters.

The dust particles generated during various processes of the fully mechanized mining face were analyzed using a Micro Particle Image Analyzer (Winner 99) to obtain images of the dust particles. The images were processed using a binarization method to extract the distribution characteristics of the dust particles, as shown in Fig. 4. Subsequently, a Laser Particle Size Analyzer (Mastersizer 3000) was employed to determine the particle size characteristics of the dust particles.

According to Fig. 4, the particle size of dust particles produced in each process of fully mechanized heading face is different. It can be seen that the dust distribution at 1 # (heading face) and 2 # (driver's position) is denser. The dust distribution at 3 # (downwind measurement of loader) and 4 # (downwind side of telescopic belt conveyor) is more dispersed than that at 1 # (heading face) and 2 # (driver's position). The dust distribution at 5 # (100 m away from heading face) and 6 # (200 m away from heading face) is more dispersed. In general, the dust concentration distribution at 2 # (driver 's position) is the densest, indicating that it poses a great threat to the health of roadheader drivers.

In the various processes of the fully mechanized working face, the particle size of dust particles mainly ranges between 6 and 8 μm. However, the diameter of breathable dust particles is consistently 7.07 μm^29^. Therefore, 8 μm is selected as the particle size threshold to effectively categorize breathable dust particles in the dust based on frequency distribution calculations. The results are presented in Table 1.

**Table 1:** Particle image analysis of each process in fully mechanized excavation face.

Process	8 μm frequency distribution (%)	≤ 8 μm cumulative distribution (%)	D_50_ (μm)
1#	12.2	40.4	11.7
2#	10.65	70.2	5.6
3#	16.5	36.8	12.2
4#	10.96	44.2	9.1
5#	12.23	48.6	8.3
6#	7.41	51.5	7.8

The 8 μm frequency distribution represents the frequency distribution value of particle sizes equal to 8 μm. The ≤ 8 μm cumulative distribution denotes the cumulative distribution of particle sizes less than or equal to 8 μm. D50 (μm) corresponds to the particle size when the cumulative frequency distribution reaches 50%.

According to Table 1, the frequency distribution of 8 μm coal dust generated at 2 # site (driver 's place) was 10.65%, and the cumulative distribution of particle size ≤ 8 μm even reached 70.2%. The cumulative distribution of particle size ≤ 8 μm in 1 # site (heading face) and 3 # site (underwind side of loader) reached 40.4% and 36.8%, but its D_50_ was 11.7 μm and 12.2 μm, indicating that the dust particle size produced by the process of 1 # site (heading face) and 3 # site (underwind side of loader) was larger, and the proportion of respirable dust produced by the other processes was about 40%. Therefore, in order to ensure the health of the driver of the fully mechanized tunneling face, it is necessary to focus on dust prevention and control at this position.

## Dust control technology of conical diversion air curtain in fully mechanized heading face

The cone-shaped diversion and air conditioning device is a kind of wall-attached effect of air flow, which changes the axial air flow supplied by the original forced air duct to the fully mechanized heading face into the rotating air flow along the roadway wall, and blows to the surrounding wall of the roadway and the whole roadway section at a certain rotating speed to form an air wall, and continuously advances to the fully mechanized heading face. Under the combined action of the axial velocity generated by the dust-containing air flow inhaled by the dust collector, a spiral linear air flow with high function is formed, and an air screen is established in front of the working area of the roadheader driver to block the outward diffusion of dust, blocking the dust generated during the operation of the roadheader. It is purified by suction into the dust collector through the dust suction duct without outflow, thus improving the dust collection efficiency of the fully mechanized excavation face.

**Figure 5:**
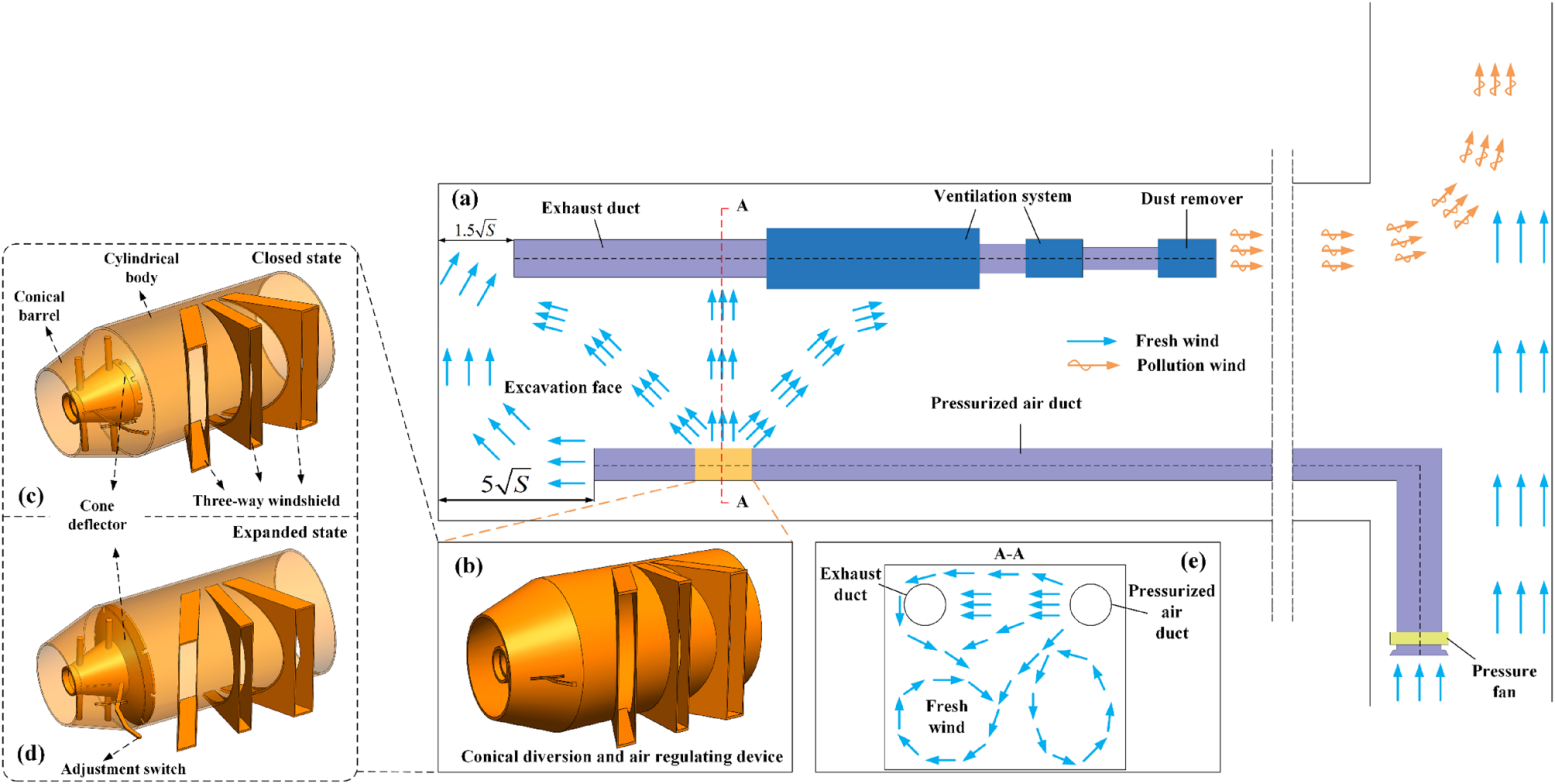
Design and installation diagram of conical diversion air-conditioning device. (**a**) Installation design of CDAC device; (**b**) The structure design of CDAC device; (**c**) The closed state of the CDAC device; (**d**) The expansion state of the CDAC device; (**e**) Cross-sectional airflow flow of the CDAC device.

The conical diversion air regulating device for fully mechanized heading face (see Fig. 5) includes a cylindrical cylinder, a conical cylinder, a conical deflector, a regulating switch, a three-way air guide cover, an air inlet and an air outlet. Among them, the three-way wind guide hood is connected with the outer wall of the cylindrical cylinder. The three-way wind guide hood is composed of three wind guide hoods in different directions. The wind guide hood near the outlet is 60° with the cylindrical cylinder, the middle wind guide hood is 90° with the outer wall of the cylindrical cylinder, and the wind guide hood near the inlet is 120° with the cylindrical cylinder. The device is installed at the outlet position of the compressed air duct in the heading face, which is connected with the outlet of the compressed air duct. The conical diversion air regulating device can be closed and expanded by adjusting the switch.

When the CDAC device is in a closed state, the internal pressure air flow of the cylindrical tube passes through the conical tube and flows out from the air outlet, as shown in Fig. 5c. When the conical diversion air-conditioning device is in the state of expansion, the conical diversion air-conditioning device forms a wind-shield surface in the cylinder body with an area smaller than the inner diameter of the cylinder body, as shown in Fig. 5d. The air flow passing through the pressure air flow in the cylinder body is effectively blocked, so that the pressure air flow flows out from the three-way air guide hood, and the pressure air flow in the pressure air cylinder is changed into a three-way dust control airflow field flowing to the fully mechanized heading face. The dust produced by the head-on cutting of the fully mechanized heading face is effectively controlled.

A hybrid ventilation system employing prolonged pressure and short suction is utilized during the commencement of the fully mechanized tunneling face. It is required that the distance between the outlet of the pressure duct and the tunneling head should not exceed $$5\sqrt S$$ (where *S* represents the cross-sectional area of the tunnel). The distance of the dust suction port from the tunneling head is calculated based on empirical formulas $$1.5$$$$\sqrt S$$$$\sqrt S$$^30^. As the face advances, the conical diversion air regulation device is deployed, directing the airflow from the side of the device towards the face, as illustrated in Fig. 5e. During face support operations, the conical diversion air regulation device is closed, directing the airflow directly toward the face. To accommodate the specific conditions of the full mechanized tunneling face, the conical diversion air regulation device is mounted on a dedicated movable device, allowing it to move synchronously with the tunneling machine and thus ensuring compliance with the layout requirements of the ventilation and dust removal system.

## Numerical simulation study on dust control of conical diversion air curtain in fully mechanized tunneling face

The flow and flow field distribution of roadway airflow directly affect the distribution of dust concentration in fully mechanized heading face. Since the 1980s, some researchers have begun to carry out experimental research on the distribution of air flow in fully mechanized heading face^31–33^. At present, with the development of computer technology, computational fluid dynamics simulation of roadway airflow and the distribution of dust particles has been widely used^20,34,35^.

### Mathematical model

In the construction process of fully mechanized tunneling face, the diffusion phenomenon of dust particles generated by the tunneling process of roadheader under the action of airflow belongs to the gas–solid two-phase flow model. In this paper, the fluid mechanics equations of particle phase based on the theory of particle phase dynamics and the conservation equations of gas phase constitute the multiphase control equations of laminar gas phase and laminar particle phase, and then simulate the diffusion process of dust particles driven by airflow in the full mechanical tunneling face. In the fully mechanized heading face, air is regarded as a continuous medium, and dust particles are regarded as discrete items. Due to a lack of reliable correlation, the model needs to pay more attention to the influence of particle-phase turbulence on the particle temperature and gas-particle drag equations. With the closure of the above model, the steady-state governing equations for three-dimensional turbulent multiphase flow in cylindrical coordinates can be expressed in a general form: convection term = diffusion term + source term. The specific mathematical models utilized are represented by Eqs. (1) to (2)^36,37^.

The general expression of the gas phase control equations is:

1 $$ \begin{aligned} \frac{\partial }{\partial x}\left( {\alpha_{g} \rho_{g} u\varphi } \right) & + \frac{\partial }{r\partial r}\left( {r\alpha_{g} \rho_{g} v\varphi } \right) + \frac{\partial }{r\partial \theta }\left( {\alpha_{g} \rho_{g} w\varphi } \right) = \frac{\partial }{\partial x}\left( {\Gamma_{\varphi } \frac{\partial \varphi }{{\partial x}}} \right) + \frac{\partial }{r\partial r}\left( {r\Gamma_{\varphi } \frac{\partial \varphi }{{\partial r}}} \right) \\ & + \frac{\partial }{{r^{2} \partial \theta }}\left( {\Gamma_{\varphi } \frac{\partial \varphi }{{\partial \theta }}} \right) + S_{\varphi } + S_{\varphi p} \\ \end{aligned} $$

The general expression of the particle phase control equations is:

2 $$ \begin{aligned} \frac{\partial }{\partial x}\left( {\alpha u_{p} \varphi_{p} } \right) & + \frac{\partial }{r\partial r}\left( {r\alpha v_{p} \varphi_{p} } \right) + \frac{\partial }{r\partial \theta }\left( {\alpha w_{p} \varphi_{p} } \right) = \frac{\partial }{\partial x}\left( {\Gamma_{\varphi p} \frac{{\partial \varphi_{p} }}{\partial x}} \right) + \frac{\partial }{r\partial r}\left( {r\Gamma_{\varphi p} \frac{{\partial \varphi_{p} }}{\partial r}} \right) \\ & + \frac{\partial }{{r^{2} \partial \theta }}\left( {\Gamma_{\varphi p} \frac{{\partial \varphi_{p} }}{\partial \theta }} \right) + S_{\varphi p} + S_{\varphi pg} \\ \end{aligned} $$

In the fully mechanized mining face, dust particles are subject to various forces such as resistance, gravity, buoyancy, Saffman force, Magnus force, thermophoretic force, adhesion force, Basset force, added mass force, and pressure gradient force. However, in this numerical simulation of airflow and particle dynamics in the mining face, the density of dust particles is much greater than that of air, and the effects of adhesion force, Basset force, and buoyancy are negligible compared to other forces. Furthermore, since the volume of dust particles in the fully mechanized mining face is less than 10% of the computational domain volume, the reactive force of dust particles on airflow can be neglected. Only the stabilizing effect of airflow on dust particles needs to be considere^2,36,38^.

This study will consider the Saffman force, Magnus force, pressure gradient force, resistance, and gravity. The Saffman force primarily refers to the force responsible for lifting particles and the pressure difference caused by the flow velocity on both sides of the dust particles, as shown in Eq. (3)^39^.

3 $$ F_{saff} = 6.46Bd_{{_{p} }}^{2} \sqrt {\rho \eta } (u_{s} - u_{p} )\frac{{\nabla u_{p} }}{{\sqrt {\left| {\nabla u_{p} } \right|} }} $$

where *B* is the experimental constant; *d*_*p*_ is the radius of the particle; *ρ* is the density of the gas phase; *η* is the viscosity of the gas phase; *u*_*p*_ is the velocity of the gas phase, *u*_*s*_ is the velocity of the solid phase; ∇ is the nabla operator.

When the fluid is in a low Reynolds number turbulence state, particles in the flow field experience rotational forces, known as the Magnus force. Assuming a rotational angular velocity of ω for the particles in the airflow, the Magnus force equation is as follows:

4 $$ F_{M} = \frac{1}{8}\pi d_{p}^{3} \rho \omega (u_{s} - u_{p} ) $$

Under typical conditions, due to the pressure gradient along the direction of gas flow in the flow field, there will be a particular force acting on the transport diffusion of spherical particles, as shown in Eq. (5).

5 $$ F_{p} = - \frac{1}{6}\pi d_{p}^{3} \frac{dp}{{dx}} $$

where *d*_*p*_/*d*_*x*_ represents the pressure gradient in the direction of gas flow.

Dust particles experience a certain resistance in the gas-phase flow field and particle field, which can be expressed as:

6 $$ F_{R} = \frac{{\rho \cdot A_{p} \cdot C_{d} }}{2}\left| {u_{s} - u_{p} } \right|(u_{s} - u_{p} ) $$

where *A*_*p*_ represents the particle's projection perpendicular to the airflow direction, and *C*_*d*_ represents the drag coefficient.

### Geometric models and parameter settings

In order to accurately numerically simulate the airflow condition, dust distribution and movement patterns in the fully utilizing the long-pressure-short-extraction ventilation method, a three-dimensional physical model of the fully mechanized mining face was constructed using three-dimensional modeling software based on the actual conditions of the 3201 mining face in Yongming Coal Mine of Shaanxi Province, The constructed physical geometry model was simplified and comprised six significant components: roadway, shearer (including body, cutting part, walking track, and scraper), blowing fan, suction fan, bridge-type shuttle car, and belt conveyor. The specific geometric model dimensions are detailed in Table 2. The positive direction of the x-axis denotes the direction from the cutting head to the end of the roadway, the positive direction of the y-axis represents the direction from one sidewall of the mining face to the other, and the positive direction of the z-axis represents the direction from the roadway floor to the roof.

**Table 2:** Geometry model parameters table.

Position	Size	Position	Size
Heading roadway	60 m × 4 m × 3.5 m	Distance between the air duct and the heading face	5 m, 10 m, 15 m, 20 m, 25 m, 30 m
Heading machine	9.7 m × 3 m × 2.1 m	Distance between the exhaust duct and the heading face	3 m
Shearer	34.3 m × 0.9 m × 0.4 m	Distance between the axis of the air duct and the tunnel wall	0.2 m
Distance between the shearer and the tunnel floor	0.7 m	Distance between the axis of the exhaust duct and the tunnel wall	0.2 m
Loader and the heading face distance	9.7 m	Distance between the axis of the air duct and the tunnel ceiling	0.6 m
Belt conveyor	25.7 m × 1.2 m × 0.5 m	Distance between the axis of the exhaust duct and the tunnel ceiling	0.6 m
Distance between the belt conveyor and the tunnel floor	0.3 m	Diameter of the conical air guide device	0.8 m
Distance between the belt conveyor and the heading face	34.3 m	Distance between the conical air guide device and the heading face	30 m
Diameter of the air duct and exhaust duct	0.6 m		

**Figure 6:**
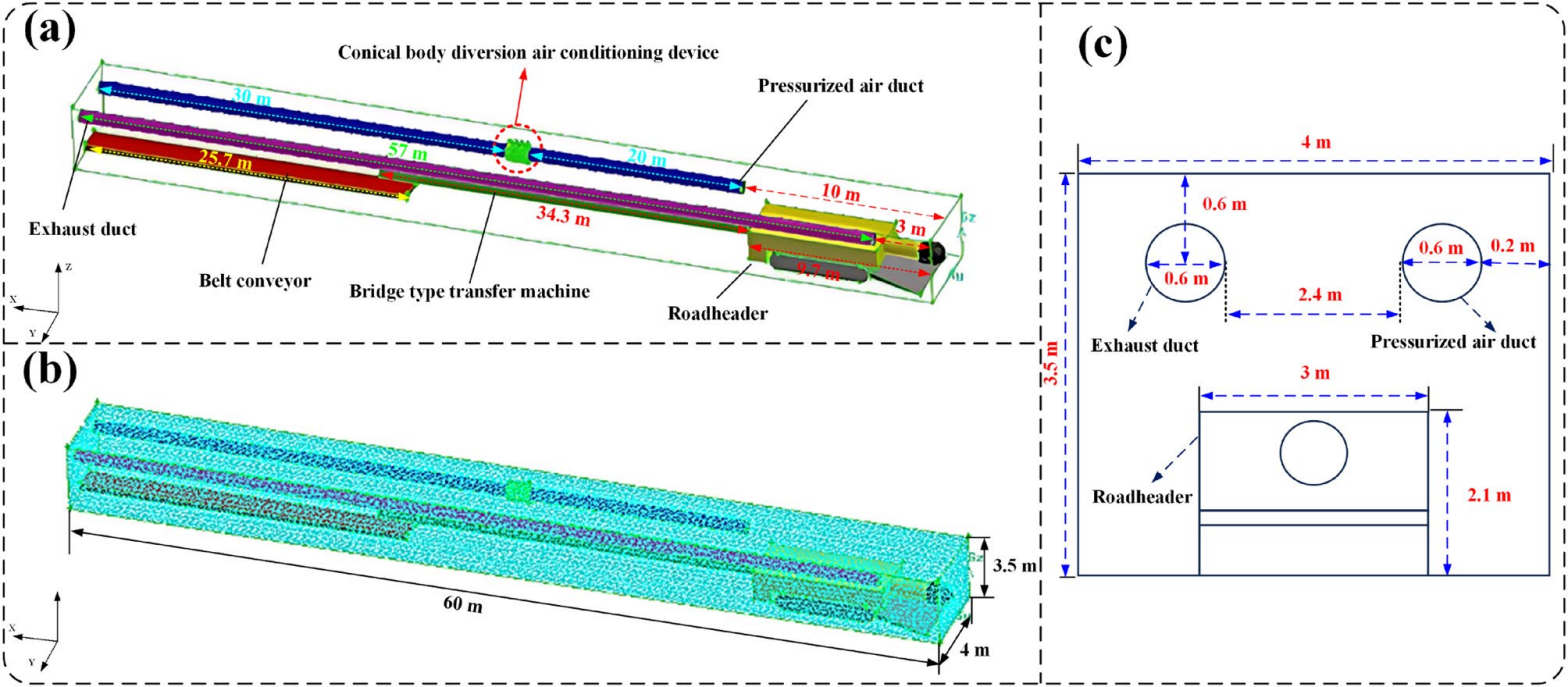
The model diagram of fully mechanized heading face. (**a**) Simulation model; (**b**) Grid division; (**c**) Transverse Interface.

Subsequently, the geometric physical model was subjected to grid partitioning using ICEM meshing software. The geometric model and grid partitioning are illustrated in Fig. 6, resulting in a total of 1,034,513 grids. The grid quality ranges from a minimum of 0.351239 to a maximum of 0.99931, with an average value of 0.916247, all meeting the requirements for simulation^2^. Finally, the grid-partitioned physical model was imported into the ANSYS Fluent solver to simulate and compute the airflow conditions, dust distribution, and movement patterns during the long-pressure-short-exhaust ventilation method employed in the fully mechanized mining face.

A cone-shaped diversion and air conditioning device is installed on the air duct of the fully mechanized tunneling face. The air conditioning ratio of the cone-shaped diversion air curtain dust control device is set to be 1:9 when the deflector is deployed. 10% of the air pressure is blown out from the axial air outlet, and 90% of the air is blown out from the air outlet of the three-way air guide cover.

### Boundary condition setting

This study mainly simulates the ventilation condition of the closed and unfolded state of the CDAC device. When the conical diversion air regulating device is in a closed state, similar to the situation when the conical diversion air regulating device is not installed, the outlets of the pressure air duct and the exhaust air duct are defined as 'Velocity_Inlet', and the end of the roadway is defined as 'Pressure_Out'. When the conical diversion air regulating device is in the unfolded state, the outlet of the pressure air duct and the outlet of the conical diversion air regulating device are defined as 'Velocity_Inlet'.

During the model setup, two assumptions were made: (1) the continuous phase airflow is treated as an ideal gas, and (2) the temperature field remains constant. In the production process of the fully mechanized mining face, the particle size range of generated particles is between 0.85 and 84.3 µm. Therefore, this simulation ignores the influence of particle shape on particle motion and adopts uniform spherical particles^2,36^. However, in the actual operation of the fully mechanized mining face, the number of generated particles is extremely large (exceeding 10e9), far exceeding the computational capacity of computer solvers. Therefore, to compute numerical simulation results within the capabilities of existing computers, a finite number of particle motions were set to represent the motion characteristics of all actual particles with specific parameters, as shown in Table 3.

**Table 3:** Main parameters in numerical simulation.

Project	Name	Settings
Solver configuration	Solver	Pressure-based
		Steady state
		Absolute velocity
		Gravity: 9.81 m/s^2^
Air properties	Density (kg/m^3^)	1.225
	Viscosity (kg/m s^−1^)	1.7894e−5
Flow model	Viscous model	k-epsilon RNG
Dust Source	Emission type	Surface
	Material	Coal
	Maximum diameter (µm)	84.3
	Minimum diameter (µm)	0.85
	Rosin–Rammler distribution index	1.93
	Particle density (g/cm^3^)	2.3
	Mass flow rate (g/min)	3 × 10^5^
Other boundary conditions	Tunnel walls	Wall

## Results and discussion

### Closure state of CDAC device

#### Air flow field

**Figure 7:**
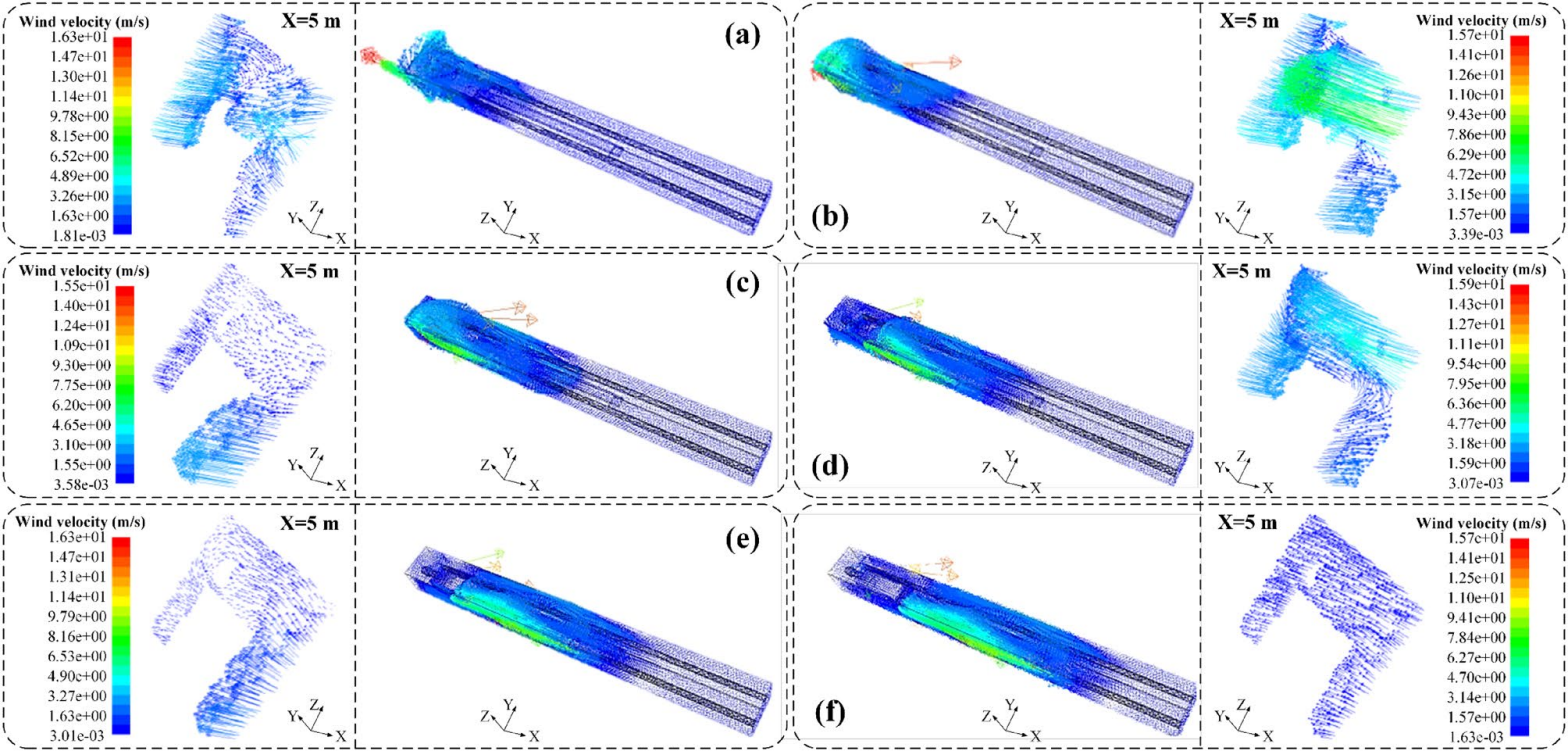
Simulation results of air flow field migration at different distances from the pressure tuyere to the head. A cross-sectional analysis was conducted at the position of X = 5 m (cutting machine operator's location). (**a**) 5 m; (**b**) 10 m; (**c**) 15 m; (**d**) 20 m; (**e**) 25 m; (**f**) 30 m.

To investigate the influence of the distance between the air inlet and the cutting head on the migration of the airflow field with the cone-shaped air diversion device in the closed state and to determine the minimum critical distance required to form an effective axial dust control airflow curtain. Considering the actual operational conditions of the fully mechanized mining face, the total air volumes of the air inlet and exhaust fan were defined as 300 m^3^/min and 240 m^3^/min, respectively. The distances of the air inlet from the cutting head were set at 5 m, 10 m, 15 m, 20 m, 25 m, and 30 m. When the cone-shaped air diversion device is closed, the situation is similar to when no cone-shaped air diversion device is installed. Therefore, the closed state of the cone-shaped air diversion device was used as the baseline for research before installation. The variation of the airflow field with different distances between the air inlet and the working face is depicted in Fig. 7.

According to Fig. 7a–f, it can be observed that as the distance between the air inlet and the mining face increases gradually from 5 to 30 m, a airflow field gradually forms flowing along the negative direction of the X-axis. With the increase in distance between the air inlet and the mining face, the axial airflow field moves further away from the mining face. When the distance between the air inlet and the mining face is between 5 and 25 m, no axial airflow field is formed, and the axial airflow field exists only at the mining face. However, when the distance between the air inlet and the mining face is 30 m, the axial airflow field begins to form, with the farthest distance from the mining face being 8.36 m, and the airflow gradually becomes more uniform. Furthermore, the minimum distance for the formation of the axial airflow field was further analyzed at the cutting machine operator's position (i.e., X = 5 m). When the distance between the air inlet and the mining face is between 5 and 25 m, the airflow field at the cutting machine operator's position is chaotic and unevenly distributed, unable to form a uniform and stable axial airflow field. However, when the distance between the air inlet and the mining face is 30 m, the airflow field at the cutting machine operator's position is relatively uniform and free from turbulent flow, with the airflow velocity decreasing from a maximum of 15.7 m/s at the air inlet to approximately 0.42 m/s. This indicates that as the distance between the air inlet and the mining face increases, the injected airflow diffuses more fully and evenly in the roadway. However, as the distance between the air inlet and the mining face increases, some of the outflowing airflow begins to lose momentum, resulting in a decrease in flow velocity and a deviation in flow direction, forming a vortex airflow field and ultimately resulting in an axial airflow field flowing along the negative direction of the X-axis.

#### Air flow-dust particle two-phase flow field

**Figure 8:**
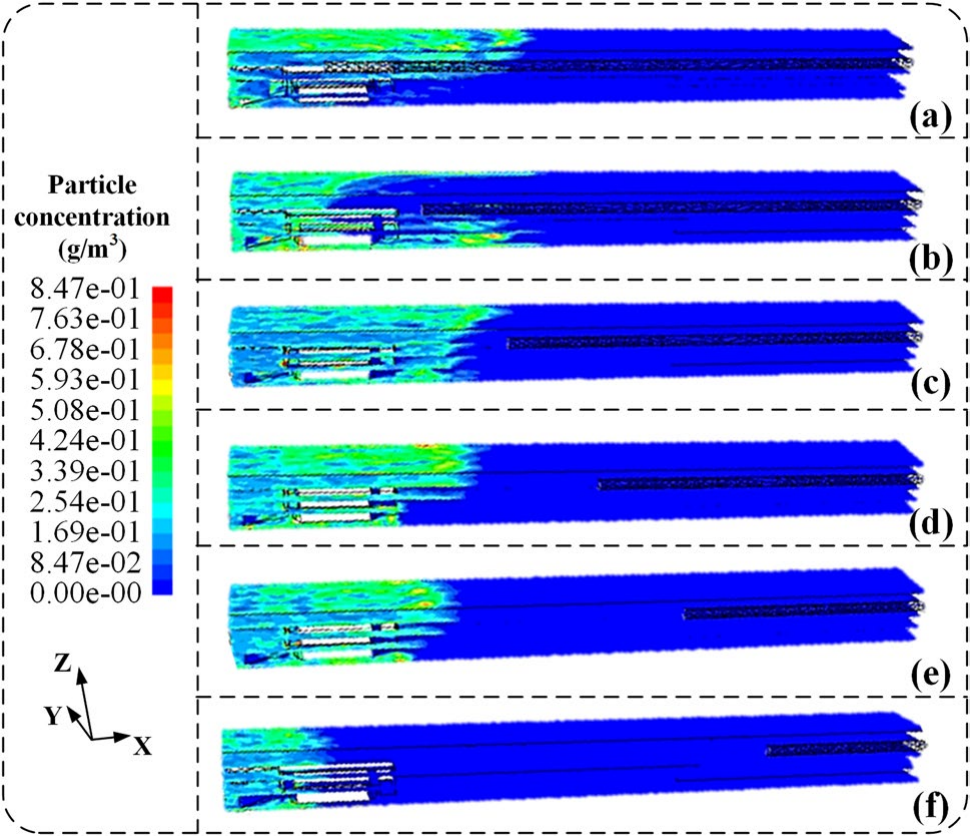
The overall diagram of the simulation results of air-borne dust flow field migration at different distances between the pressure tuyere and the tunneling head. (**a**) 5 m; (**b**) 10 m; (**c**) 15 m; (**d**) 20 m; (**e**) 25 m; (**f**) 30 m.

Based on Fig. 8a–f, it can be observed that as the distance between the air inlet and the mining face increases, the aggregation range of dust particles tends to decrease. When the distance between the air inlet and the mining face is 5 m, the diffusion distance of dust particles with concentrations above 50 mg/m^3^ is 16.5 m. As the distance increases to 25 m, the diffusion distance gradually decreases to 11.2 m. When the distance reaches 30 m, the diffusion distance sharply decreases to 5.8 m for dust particles with concentrations above 50 mg/m^3^. Further analysis at the cutting machine operator's position (X = 5 m) reveals that within the range of 5–25 m between the air inlet and the mining face, the dust concentration fluctuates between 162.5 and 237.8 mg/m^3^. When the distance between the air inlet and the mining face is 30 m, the dust concentration decreases to 129.8 mg/m^3^. This indicates that airborne dust transport simulation results are consistent with airflow field simulations. The minimum distance between the air inlet and the mining face required to form an axial airflow field is 30 m. At this point, an airflow curtain flows along the negative X-axis direction, continuously reducing the diffusion distance of dust particles with concentrations above 50 mg/m^3^, effectively controlling the movement of dust particles to maintain the axial airflow field. However, the cutting machine operator's position remains in an environment with relatively high dust concentration, posing a severe health risk to the operator.

### Model validation

**Figure 9:**
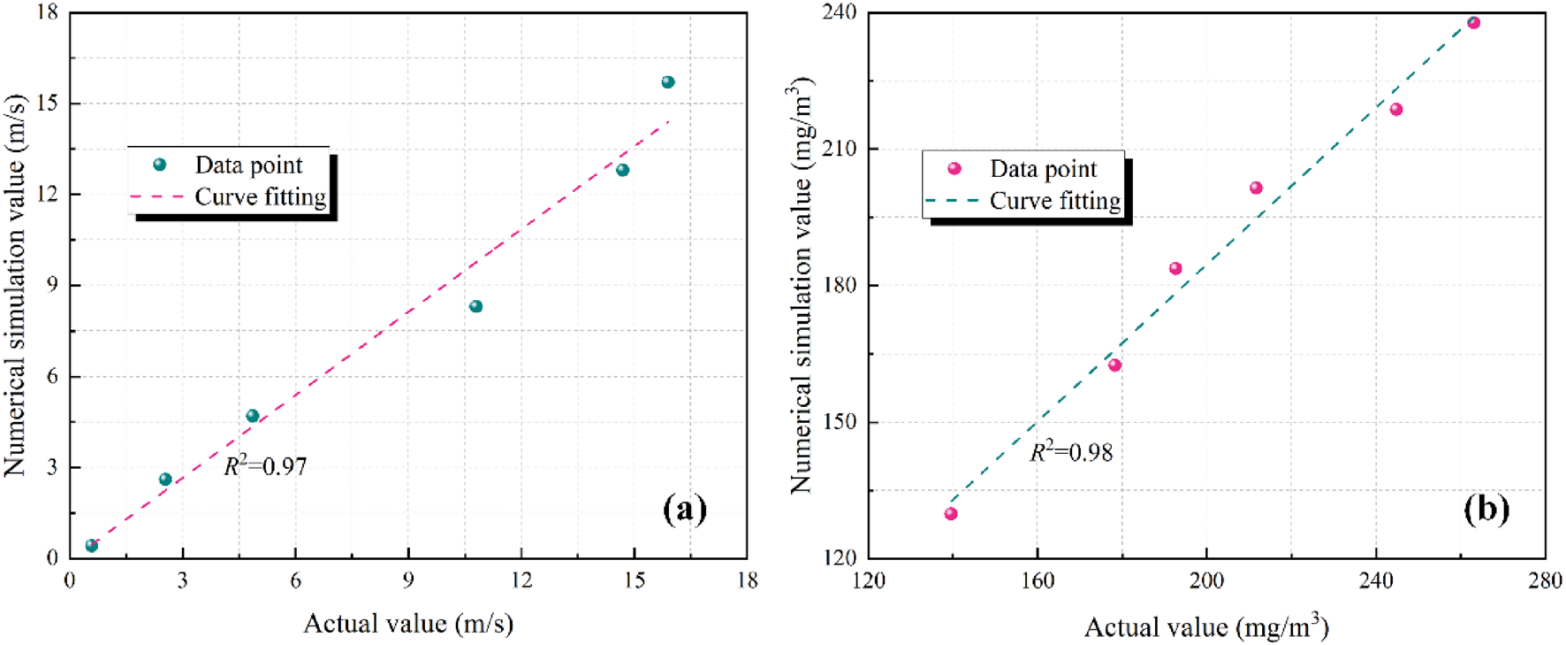
Curve fitting of numerical simulation and experimental results for airflow and particle field. (**a**) Airflow field; (**b**) particle field.

To validate the feasibility of the constructed geometric model and boundary conditions, measurements were taken at the 3201 mining face of Yongming Coal Mine. In the 3201 mining face, the air supply duct had a supply volume of 300 m^3^/min, and the distance from the air supply opening to the face of the mining face was set at 5, 10, 15, 20, 25, and 30 m, respectively. The exhaust duct had an exhaust volume of 240 m^3^/min, and the distance from the exhaust opening to the face of the mining face was set at 3 m. The operator position of the cutting machine was selected as the measurement point. The airflow at this position was measured using a handheld air velocity sensor (TSI8455), while the dust concentration was measured using an explosion-proof direct-reading mine dust detector (CCZ-1000). The dust concentration at a distance of 5 m from the face was taken as the source of dust concentration, as shown in Fig. 9.

According to Fig. 9a,b, it can be observed that at the position of the cutting machine operator, the results obtained from numerical simulations exhibit a high degree of fit with the experimentally measured results. The coefficient of determination *R*^2^ for the airflow field is 0.97, and for the particle field is 0.98. This indicates that the numerical simulation results are consistent with the experimental measurements for airflow and particle fields. However, due to various interfering factors affecting the experimental data, there is a certain degree of error between the numerical simulation results and the experimental measurements. The relative error range for the airflow field is 1.25–14.14%, and for the particle field is 4.67–10.66%. As the maximum relative error range for both fields is less than 15%^2^, the numerical simulation results of this study are considered accurate, providing valuable data support for investigating the airflow and particle fields when the conical diversion adjustment device is deployed.

### Expansion state of CDAC device

The conical diversion adjustment device is deployed in the expanded state to better reduce particle concentration at the cutting machine operator's position. This divides the axial airflow in the pressure duct into 10% axial airflow and 90% radial airflow, forming a radial airflow field to lower the particle concentration at the cutting machine operator's position. Therefore, based on the airflow field distribution characteristics, when the conical diversion adjustment device is closed, the radial air outlet of the conical diversion adjustment device is set at a distance of 30 m from the cutting face. To meet the ventilation requirements at the cutting machine operator's position, a ventilation duct of 20 m in length will be installed at the axial air outlet of the conical diversion adjustment device to supply air.

The airflow rate is a crucial factor influencing the formation of radial air curtains by the conical diversion adjustment device. Appropriate airflow rates can stabilize the air curtain and achieve optimal dust prevention performance. Considering the actual operating conditions of the fully mechanized mining face, based on the numerical simulation results when the conical diversion adjustment device is closed, we investigate the airflow and particle fields with different airflow rates in the pressure duct when the conical diversion adjustment device is deployed. The airflow rates in the pressure duct are set to 200 m^3^/min, 250 m^3^/min, 300 m^3^/min, 500 m^3^/min, and 600 m^3^/min, while the total airflow rate of the exhaust duct is set to 240 m^3^/min.

#### Air flow field

**Figure 10:**
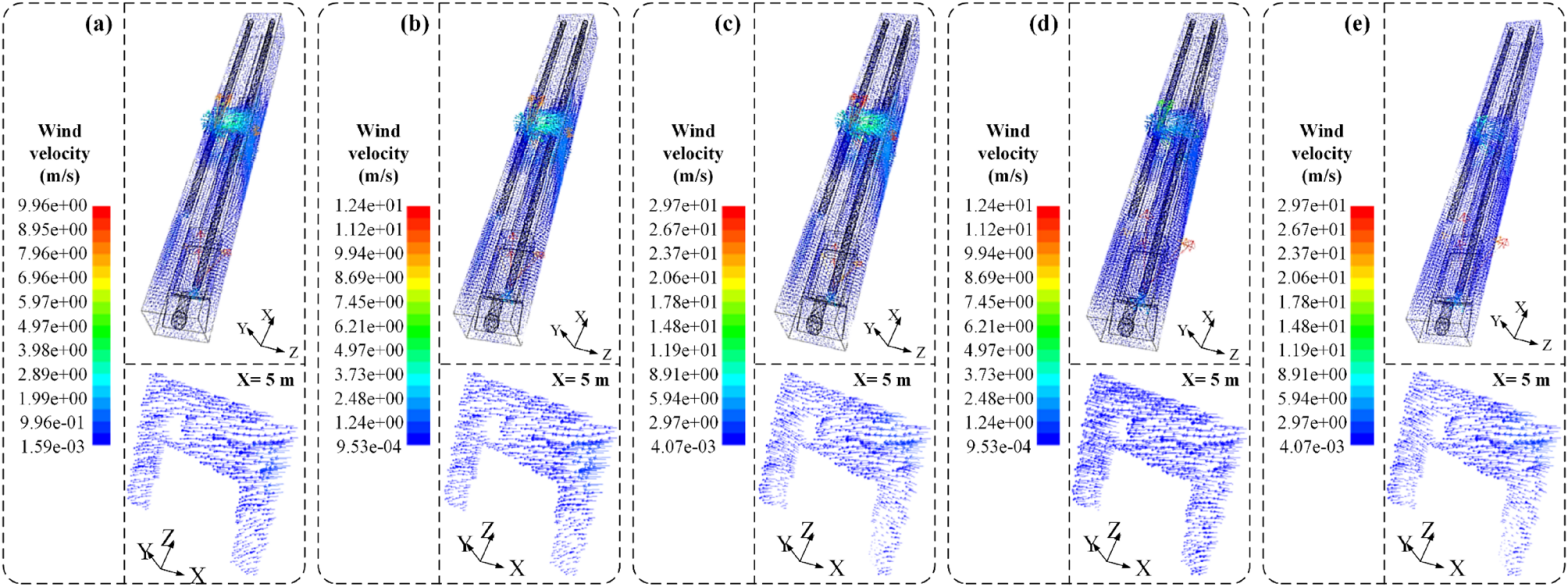
The overall diagram of the simulation results of the flow field migration of different pressure air volume after the expansion of the CDAC device. (**a**) 200 m^3^/min; (**b**) 250 m^3^/min; (**c**) 300 m^3^/min; (**d**) 400 m^3^/min; (**e**) 600 m^3^/min.

According to Fig. 10a–e, it can be observed that with the gradual increase in the airflow rate, the stability of the radial airflow field initially increases and then decreases. When the airflow rate is 200 m^3^/min, the airflow velocity at the radial air outlet of the device is 9.76 m/s. When the airflow rate is increased to 250 m^3^/min, the airflow velocity at the radial air outlet of the device increases to 12.34 m/s. As the airflow rate increases to 300 m^3^/min, the airflow velocity at the radial air outlet reaches 19.21 m/s. However, when the airflow rate is increased to 400 m^3^/min and 600 m^3^/min, the airflow velocity at the radial air outlet decreases to 11.85 m/s and 5.43 m/s, respectively. With the increasing airflow rate, it becomes more difficult to form a uniformly controlled dust airflow field along the negative X-axis direction at the section where the cutting machine operator is located (X = 5 m). When the airflow rates are 200 m^3^/min, 250 m^3^/min, and 300 m^3^/min, a controlled dust airflow field can be formed along the negative X-axis direction. However, when the airflow rate increases to 600 m^3^/min, in the area 0–0.8 m above the bottom of the section where the cutting machine operator is located, the airflow is reduced, and some airflow flows towards the sidewalls of the tunnel, failing to form a uniformly controlled dust airflow field along the negative X-axis direction.

This indicates that after the conical diversion adjustment device is deployed, 90% of the airflow through the radial air outlet forms an impact jet, covering the sidewalls of the tunnel and forming a wall-attached dust control radial airflow field. The remaining 10% of the airflow discharged through the axial air outlet, due to its similar velocity to the surrounding airflow, does not create a suction effect with the surrounding air, allowing the airflow to continue flowing along the negative X-axis direction and spreading towards the sidewalls of the tunnel with the exhaust duct. However, as the airflow rate increases, the high-speed airflow discharged through the axial air outlet creates an impact jet on the cutting face, resulting in turbulent airflow in the tunnel.

#### Air flow-dust particle two-phase flow field

**Figure 11:**
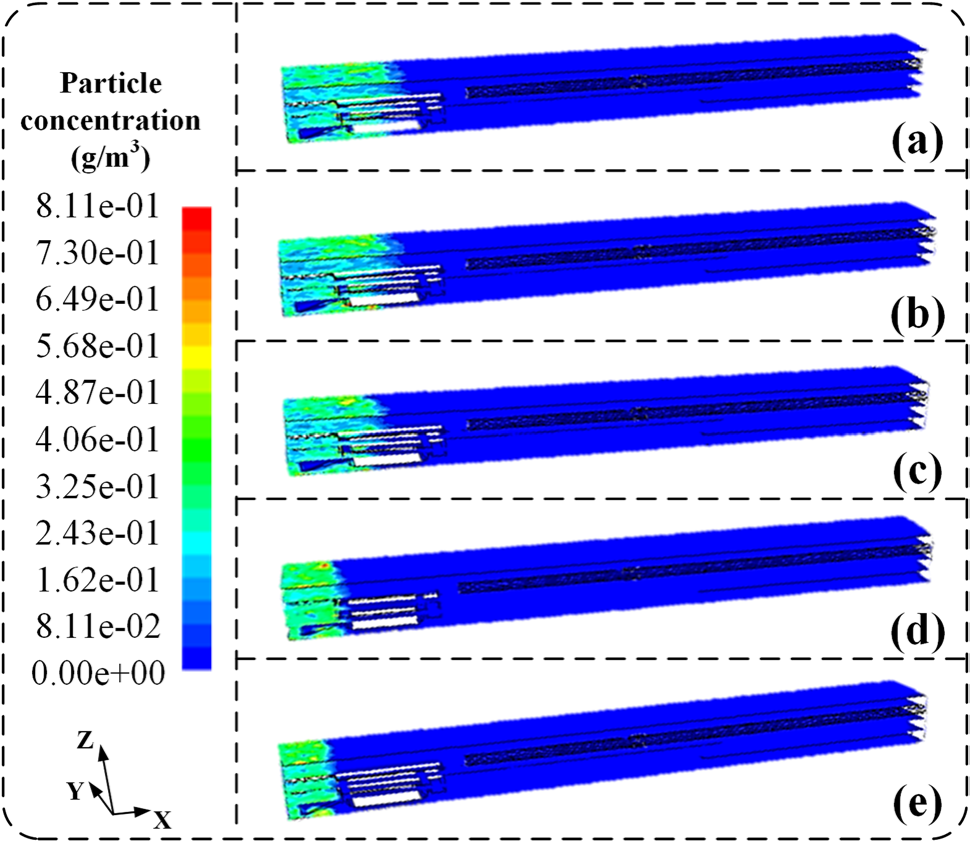
The overall diagram of the simulation results of the flow field migration of airborne dust with different pressure air volume after the deployment of the conical diversion air regulating device. (**a**) 200 m^3^/min; (**b**) 250 m^3^/min; (**c**) 300 m^3^/min; (**d**) 400 m^3^/min; (**e**) 600 m^3^/min.

After deploying the conical diversion adjustment device, simulations were conducted to investigate the dispersion of airborne dust particles under different airflow rates, as illustrated in Fig. 11. As shown in Fig. 11a–e, with the increase in airflow rate, the dispersion distance of high-concentration dust particles (> 50 mg/m^3^) gradually decreases. For instance, when the airflow rate is 200 m^3^/min, the dispersion distance of high-concentration dust particles is 6.8 m. As the airflow rate increases to 250 m^3^/min, the dispersion distance decreases from 6.8 to 5.6 m. Further, at an airflow rate of 300 m^3^/min, the dispersion distance reduces to 5.0 m. Similarly, at higher airflow rates of 400 m^3^/min and 600 m^3^/min, the dispersion distances decrease to 3.3 m and 3.2 m, respectively. At the driver's location (X = 5 m), the dust concentration also decreases with increasing airflow rate. For example, at an airflow rate of 200 m^3^/min, the dust concentration is approximately 201.5 mg/m^3^. However, as the airflow rate increases to 300 m^3^/min, the dust concentration sharply decreases to 18.8 mg/m^3^. Similarly, at 400 m^3^/min airflow rates and 600 m^3^/min, the dust concentration gradually decreases to 18.3 mg/m^3^ and 18.1 mg/m^3^, respectively.

This indicates that changes occur in the airflow field at the mining face after deploying the conical diversion adjustment device. A radial dust control airflow field is formed at the radial air outlet position, maintaining the dispersion distance of high-concentration dust particles near the cutting machine operator. Due to the diversion of 10% of the airflow through the axial air outlet, when the airflow rate is relatively low, the dispersion distance of high-concentration dust is shorter, leading to a high dust concentration environment around the cutting machine operator. However, at the critical airflow rate, the formation of an axial airflow field along the negative X-axis direction leads to a sharp decrease in the dust concentration near the cutting machine operator. When the airflow rate exceeds the critical airflow rate, the axial airflow continuously impacts the mining face, effectively reducing the dust concentration near the cutting machine operator by aggregating high-concentration dust particles.

In summary, after deploying the conical diversion adjustment device, the airflow rate controls the formation of both axial and radial airflow fields. At an airflow rate of 300 m^3^/min, the airflow field is relatively stable and uniform, with the maximum radial airflow velocity, forming a stable radial airflow curtain. This effectively impedes the dispersion movement along the positive X-axis direction. The axial airflow curtain formed by the axial airflow field controls the dust particle concentration near the cutting machine operator, ensuring the operator's health and safety.

## Conclusions

In order to effectively control the dust concentration of the fully mechanized heading face and directly protect the health of the driver of the roadheader, this paper studies the physical and chemical characteristics of the dust in each process of the heading face, and proposes a cone-shaped diversion air curtain dust control technology. The numerical simulation is used to explore the dust control ability of the cone-shaped diversion air curtain. The specific conclusions are as follows:

1. The particle size of dust particles produced in each process of fully mechanized heading face is different. The dust concentration at the driver’s place is large, and the cumulative distribution of particle size ≤ 8 μm is as high as 70.2%. Therefore, it is necessary to focus on the prevention and control of this location.
2. Based on the wall-attached effect of airflow, a conical diversion and air-regulating device is designed, which can change the axial airflow in the roadway of fully mechanized heading face into a rotating airflow along the roadway wall, and blow it to the surrounding wall of the roadway and the whole roadway section at a certain rotation speed. An air screen that can block the outward diffusion of dust is established in front of the working area of the roadheader driver.
3. CFD is used to simulate the airflow field and the two-phase flow field of airflow particles when the cone-shaped air-conditioning device is closed and displayed. It is determined that the conical diversion air regulating device is installed at the pressure air outlet, and the distance between the pressure air outlet and the heading head is 30 m, which can better form the air screen. When the conical diversion air regulating device is in the unfolded state, with the increase of the compressed air volume, the diffusion distance of high concentration dust decreases continuously, and the dust concentration at X = 5 m driver also decreases gradually. When the optimal pressure air volume is 400 m^3^/min, the air curtain barrier can be better formed to block the dust particles.

## Data Availability

The datasets used and/or analysed during the current study available from the corresponding author on reasonable request.
